# Selenium and Selenoproteins in Health

**DOI:** 10.3390/biom13050799

**Published:** 2023-05-08

**Authors:** Fan Zhang, Xuelian Li, Yumiao Wei

**Affiliations:** 1 Department of Cardiology, Union Hospital, Tongji Medical College, Huazhong University of Science and Technology, Wuhan 430022, China; 2 Hubei Key Laboratory of Biological Targeted Therapy, Union Hospital, Tongji Medical College, Huazhong University of Science and Technology, Wuhan 430022, China; 3 Hubei Engineering Research Center for Immunological Diagnosis and Therapy of Cardiovascular Diseases, Union Hospital, Tongji Medical College, Huazhong University of Science and Technology, Wuhan 430022, China

**Keywords:** selenium, selenoprotein, oxidative stress, immune system, brain function, cardiovascular disease, cancer, type 2 diabetes, heavy metals

## Abstract

Selenium is a trace mineral that is essential for health. After being obtained from food and taken up by the liver, selenium performs various physiological functions in the body in the form of selenoproteins, which are best known for their redox activity and anti-inflammatory properties. Selenium stimulates the activation of immune cells and is important for the activation of the immune system. Selenium is also essential for the maintenance of brain function. Selenium supplements can regulate lipid metabolism, cell apoptosis, and autophagy, and have displayed significant alleviating effects in most cardiovascular diseases. However, the effect of increased selenium intake on the risk of cancer remains unclear. Elevated serum selenium levels are associated with an increased risk of type 2 diabetes, and this relationship is complex and nonlinear. Selenium supplementation seems beneficial to some extent; however, existing studies have not fully explained the influence of selenium on various diseases. Further, more intervention trials are needed to verify the beneficial or harmful effects of selenium supplementation in various diseases.

## 1. Introduction

Trace elements play an important role in maintaining fundamental physiological functions [1]. Selenium (Se) is a trace element first discovered in 1817 by the Swedish chemist, Jöns Jacob Berzelius. Originally, selenium was considered a naturally occurring toxicant [2]; however, this view changed following the unexpected discovery that selenium prevented liver necrosis in rats by Schwarz and Foltz in 1957 [3]. Since then, the perception of selenium as a health threat has changed. In fact, selenium began to be viewed as an element beneficial to health. Selenium from food exerts its physiological role by co-translationally incorporating many proteins as components of the amino acid selenocysteine (Sec) [4]. Notably, selenium provides selenoprotein molecules with a range of redox properties that maintain redox homeostasis [5,6].

## 2. Selenium Intake

The total amount of selenium in humans is approximately 3–20 mg. As an essential mineral micronutrient, selenium is mainly obtained from foods, such as cereals, meat, fish, and eggs [7]. Generally, selenium concentrations vary from food to food, and animal-based foods > vegetables > cereals > fruits. Cereals are the main source of selenium; however, their selenium content is relatively low, ranging from 0.01 to 0.55 μg/g. The selenium content is between 0.08 and 0.7 μg/g in animal-based foods and less than 0.1 μg/g in vegetables and fruits. Brazil nuts are the most abundant source of dietary selenium, with selenium levels up to 512 g/g [8]. Soil is the main source of selenium in plants. Soil total selenium content below 0.1 mg/kg is considered selenium deficient, 0.2–0.3 mg/kg is generally selenium deficient, and more than 0.4 mg/kg is selenium enriched [9]. The bioavailability of plants to different forms of selenium in soil varies, and selenate > organic selenium > selenite > selenium > selenide. In addition, the uptake of selenium by plants is also influenced by soil pH, redox conditions, microbial activity, and organic matter, but these aspects will not be covered in detail here. The World Health Organization (WHO) recommends a selenium intake level of 55 µg/day for adults, with the tolerable upper limit set at 400 μg/day. Moderate selenium intake and a balanced diet are critical to maintain.

The form of selenium in food affects its absorption by humans. Generally, dietary selenium exists as organic selenium compounds, selenate, and selenite, with a bioavailability of 70–95% [10,11]. Selenoamino acids often have higher bioavailability than inorganic selenium [12], and selenium in plant foods is more bioavailable than that in animal foods [11]. Selenomethionine (SeMet), the chief nutritional form of selenium, cannot be synthesised by higher organisms. The synthesis of SeMet relies on plants and fungi [13]. Approximately 90% of the selenium in plants is present as SeMet. The bioavailability can reach 95–98% [14,15]. Selenocysteine (Sec) is another organic selenium compound derived mainly from animal foods. Inorganic selenium mainly accumulates in plants via the sulphur assimilation pathway but is also present in water. Selenates and selenites ingested by humans are eventually converted to SeMet.

## 3. Selenoproteins

Selenium from food is taken up and present in humans in the natural organic forms of selenocysteine and selenoprotein. They are stored in different organs and tissues: 30% in liver, 30% in muscle, 15% in kidney, 10% in plasma, and 15% in other organs [16]. The selenium concentration in liver reflects intestinal absorption levels. The liver synthesises selenoprotein P (SELENOP), which enters the bloodstream and supplies selenium to other tissues and organs [17,18,19]. The biological effects of selenium are primarily mediated by selenoproteins [20]. Almost all selenoproteins contain single Sec residues at their enzyme active sites [21], which are essential for their activity. Sec, the 21st naturally occurring, genetically coded amino acid, is a sulphur-to-selenium substituted variant of cysteine (Cys) [22]. Selenium and sulphur belong to the same group. Therefore, Sec and Cys exhibit similar chemical properties and participate in similar chemical reactions [23]. However, compared with Cys, Sec has higher nucleophilicity [24], oxidation susceptibility, and acidity, which is mainly reflected in its relatively lower pKa (5.2) [25,26]. Therefore, most side-chain selenols can be deprotonated at biological pH, and Sec is reactively superior to Cys [27,28].

**Figure 1:**
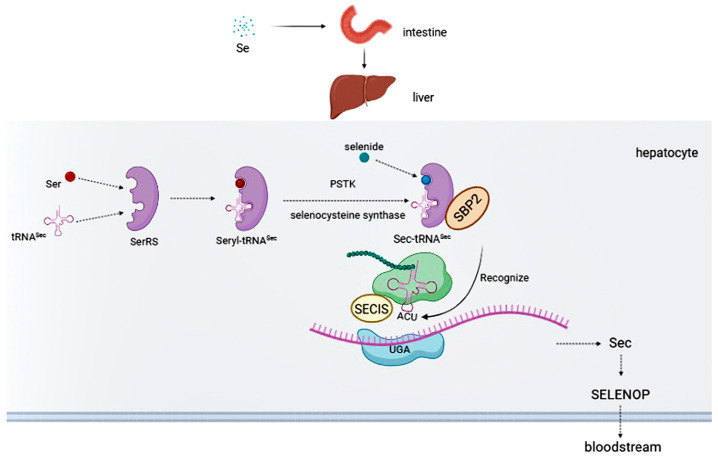
Selenium from food is transformed in the liver and inserted into polypeptide chains, and the liver synthesises SELENOP to supply selenium to the whole body. Selenide is first phosphorylated, then transferred to the ribosomal A site under the recognition of SBP2, and finally recognised by the UGA codon under the decoding action of SECIS.

In humans, Sec is the only naturally occurring amino acid that lacks a cognate aminoacyl-tRNA synthetase. Thus, Sec requires a specific biosynthetic pathway [29] (Figure 1). First, under the catalysis of seryl-tRNA synthetase (SerRS), selenocysteine-specific tRNA (tRNA^Sec^) binds with Ser to form Seryl-tRNA^Sec^, which is subsequently converted to Sec-tRNA^Sec^ by O-phosphoseryl-tRNA^Sec^ kinase (PSTK) and selenocysteine synthase [30,31]. Sec-tRNA^Sec^ can ligate to Sec insertion sequence-binding protein 2 (SBP2), which specifically recognises the stop codon UGA [32,33]. This process requires the participation of the Sec insertion sequence (SECIS) element in the 3’ untranslated region of the mRNA to decode the UGA codon as Sec [34,35]. Finally, under the interaction of SECIS and SBP2, Sec is delivered to the ribosome and co-translationally inserted into nascent polypeptide chains [20].

A total of 25 selenoprotein genes have been described in humans, and most of these genes encode redox enzymes [36,37]. Selenoproteins are distributed in different organs and tissues, and have different substrate specificities and functions (Table 1). The glutathione peroxidase (GPX) and thioredoxin reductase (TxnRd) families are involved in cellular antioxidative defense systems and the maintenance of intracellular redox states to maintain cell viability [38,39] (though see below). These families often function in parallel in humans. The three iodothyronine deiodinases (Dio1,2,3) are selenoproteins with developmental-, cell-, and pathology-related expression patterns. Dio1 and Dio2 participate in the production of the active thyroid hormone T3 [40], while Dio3 contributes to the generation of the inactive rT3 and T2. Human SELENOP is a monomeric glycoprotein containing 10 selenocysteine residues, an important feature that distinguishes it from other selenoproteins. Therefore, SELENOP, a selenium transport protein, accounts for approximately 40% of the total selenium concentration in human plasma and can bind to specific receptors on cell membranes to deliver selenium to other cells, such as low-density lipoprotein receptor-related protein 8 (LRP8) and megalin receptors on kidney proximal tubule epithelial cells [17,41]. SELENOP also has redox properties and is associated with the protection of endothelial function. SELENOP binds heparin and participates in insulin resistance. Selenoprotein S is associated with inflammatory responses and endoplasmic reticulum stress [42,43]. Selenoproteins W (SELENOW) and N participate in muscle development and maintenance [44,45]. However, the functions of many selenoproteins have not been fully elucidated and require further investigation. Selenium functions as a redox centre in these selenoproteins. Therefore, a deficiency in the trace element selenium can cause several disorders.

## 4. Health Effects of Selenium and Selenoproteins

The unique biological characteristics of the trace mineral selenium make it indispensable for health. Although selenium is present at very low levels in the human body, selenium deficiency can cause dysfunction in various systems. Selenium cannot be synthesised in the human body and is mainly obtained from food. The physiological function of selenium is mainly reflected in selenoproteins, which have excellent efficacy in resisting oxidative stress, inflammation, and other adverse factors. Appropriate supplementation of selenium can not only activate the immune system but also affect brain function, cardiovascular diseases (CVDs), cancer, and heavy metal-based illness. Although studies have suggested that high selenium levels have negative effects on some specific diseases such as type 2 diabetes mellitus (T2DM), further exploration of selenium is still beneficial and may provide new ideas for the treatment of various diseases.

### 4.1. Oxidative Stress

Redox homeostasis is the basis for maintaining life activities. Oxidative stress manifests as an imbalance between the cellular oxidative and antioxidant systems. This imbalance is mainly reflected in the production of large amounts of reactive oxygen species (ROS) that exceed the scavenging capacity of antioxidant defense systems, ultimately leading to structural and functional damage to DNA, lipids, and proteins. The mitochondria are considered a major source of ROS, and excessive ROS can cause structural damage to the mitochondria. Hydroperoxides, especially hydrogen peroxide (H_2_O_2_), serve as the major ROS in redox regulation, and are responsible for cell signaling, enzymatic reactions, energy metabolism, and cell cycle. However, superabundant hydroperoxides result in unspecific proteins oxidation and biomolecules damage. The removal of hydroperoxides relies on efficient reducing systems. Selenium acts as a critical antioxidant in affecting various tissues and cells and contributes to the removal of ROS, especially the removal of hydroperoxides. This effect has been reported in the heart [110], liver [111], kidneys [112], thyroid [113], and brain [114] (the mechanism is described later). In addition, the antioxidant effect of selenium may be responsible for its resistance to inflammation, apoptosis, and autophagy.

The defense of selenium on ROS is primarily mediated by selenoproteins, which have redox activity and can catalyse the reduction of hydroperoxides by thiols; however, some differences exist in their substrate specificity. The best known are the GPXs and the thioredoxin (Trx) system, which are the main members of the antioxidant system. Five of the eight human GPXs are selenoproteins, and their active site contains a Sec, while the active site of other three is cysteine. However, the catalytic efficiency of GPXs is conspicuous irrespective of their substrate or active site. The active site of GPXs contains a conserved tetrad formed by peroxidatic Sec, glutamine, tryptophan, and asparagine [115]. The Sec residue can be oxidised by hydroperoxides and forms selenenic acid or a selenenylamide intermediate. These intermediates will be reduced back to selenate soon by thiol [116]. Due to the high reactivity of Sec residues, GPXs react on H_2_O_2_ with high second-order rate constants, which helps to reduce the cellular H_2_O_2_ concentration. GPX1 was the first mammalian selenoprotein to be identified and is the most abundantly expressed GPX. GPX1 is present in the mitochondria and cytoplasm. GPX1 utilizes glutathione (GSH) to reduce hydroperoxides and is highly sensitive to selenium levels. GPX2 has similar substrate specificities to GPX1 and is mainly found in the mucosa of the gastrointestinal tract and various endothelial cells [117]. GPX2 maintains mucosal homeostasis and regulates intestinal regeneration. The expression of GPX2 was only found to be downregulated in patients with severe selenium deficiency [118]. GPX3 is an extracellular glycoprotein that accepts the oxidation of GSH, Trx, and glutaredoxin and is present at high levels in white and brown adipose tissues [119]. GPX4 is found to be the only isoform which can decrease phosphatidylcholine hydroperoxide [120]. It is present in the mitochondria and cytoplasm; however, only the mitochondrial form of GPX4 protects cells from oxidative stress. GPX6 is a close homologue of plasma GPX3 and is expressed in the embryonic and olfactory systems. Not enough is known about GPX6 yet.

The thioredoxin system is composed of nicotinamide adenine dinucleotide phosphate (NADPH), Trx, and thioredoxin reductase (TXNRD). Trx and TXNRD provide a coupled redox system required for redox reactions. The thioredoxin system prevents oxidative damage to cells and maintains redox homeostasis. Trx exerts its antioxidant activity by transferring electrons to thioredoxin peroxidases and reducing oxidised Cys disulphide or Cys-SOH in proteins to thiols. Trx can also participate in shaping intracellular H_2_O_2_ gradients. TXNRD belongs to the pyridine nucleotide disulphide oxidoreductase family of enzymes and can reduce the oxidised form of Trx using NADPH as a co-substrate. Three TXNRD isoforms have been identified in mammals: cytosolic (TXNRD1), mitochondrial (TXNRD2), and thioredoxin glutathione reductase (TXNRD3). The Trx system performs its antioxidant functions by reducing methionine sulfoxide reductases and ribonucleotide reductases. This system also regulates the activity of redox-sensitive transcription factors (especially AP-1 and NF-κB). The antioxidant properties of the Trx system indicate its vital contribution to the antioxidant defense system and the maintenance of cell homeostasis.

### 4.2. Immune System

**Figure 2:**
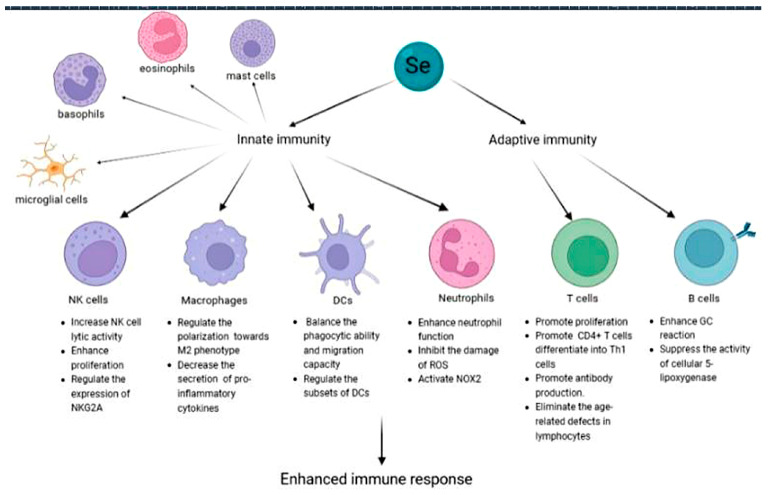
The role of selenium in the immune system. Selenium regulates the viability of NK cells, macrophages, DCs, granulocytes, mast cells, and microglia in innate immunity. Selenium affects the proliferation and differentiation of T cells, and regulates B cell differentiation and viability by affecting Tfh cells and 5-lipoxygenase activity.

Numerous studies have suggested that selenium supplementation enhances the immune response to various harmful conditions. Selenium supplementation is involved in both innate and adaptive immunity [121,122,123,124]. Based on existing research, the selenium supplement is mainly considered as an immunomodulator as it has regulatory effects on various immune cells [125,126,127] (Figure 2). Prior results provide important insights into the mechanisms by which selenium affects immunity.

#### 4.2.1. Innate Immunity

The immunostimulatory effect of selenium is applicable even in individuals with sufficient selenium. A daily supplement of 200 μg selenium to selenium-replete US patients for 8 weeks increased the lytic activity of NK cells, as selenium could regulate the expression of NK cell inhibitory receptor CD94/Natural Killer G2A (NKG2A) [128]. Compared with baseline, individuals replenished with selenium showed an 82.3% increase in NK cell activity [127,129]. Selenium exhibits special regulation on macrophages. Mice fed with both selenium-enriched and selenium-deficient diets were found to have a faster resolution of inflammation in the Se-enriched group. Macrophages can be activated and eventually differentiate into the classical M1 and the alternative M2. Selenium regulates the polarization of macrophages towards the anti-inflammatory M2 phenotype, and reduce the pro-inflammatory M1 phenotype. Selenium can also decrease the secretion levels of pro-inflammatory cytokines such as iNOS, IL-1β, IL-10, PTGe, and NF-Κb [130]. The effects of selenium on dendritic cells (DCs) are multifaceted. A mediate selenium supplement contributes to keeping balance between phagocytic ability and migration capacity of immature DCs. It also contributes to the chemotactic migration of mature DCs. Selenium regulates the subsets of DCs. It is reported that a selenium supplement is able to reduce the proportion of activated DCs while increasing tolerogenic DCs [126,131]. As a constituent of selenoproteins, selenium is integral for the proper functioning of neutrophils. Their effects depend to a great extent on their ability to activate membrane-associated nicotinamide adenine dinucleotide phosphate oxidase 2 (NOX2), which is essential for the microbicidal activity of neutrophils [132]. Selenoproteins, especially GPX1, are probably involved in the regulation of ROS-dependent neutrophil extracellular traps (NETs) through affecting cytoplasmic and mitochondrial ROS accumulation [122]. Selenium deficiency was reported to impair the bacterial killing ability of mouse neutrophils during in vitro tests [133]. These things considered, selenium could protect mast cells, eosinophils, and basophils from ROS [125], thus regulating their proliferation, differentiation, and recruitment [134]. It was found to reduce mast cell infiltration in ischemia-reperfusion injury [135,136]. Mast cells pre-treated with selenium selenite are related to less mediator release [137]. Regression of eosinophilic enteritis and eosinophilia was reported in selenium-deficient rats when fed a selenium-supplemented diet for 4–5 weeks [138]. Basophils in selenium-deficient rats have 35% of control of phospholipid hydroperoxide GPXs activity and <1% of control of GPXs activity. A selenium supplement helps to reverse these changes [139]. Selenium eliminates the ROS-induced microglial cells migration [140]. It increases the GPXs and TXNRD levels to prevent the transcription of pro-inflammatory cytokines such as IL-1ß and iNOS [141]. 

#### 4.2.2. Adaptive Immunity

T cell selenoprotein-deficient mice displayed moderate to severe atrophy of the lymph nodes, thymus, and spleen, with a 50–80% reduction in cellularity [142]. Generally, TCRs are coupled to multiple intracellular signaling molecules, and T cells are stimulated by TCR/CD3 complexes [143,144]. This process is accompanied by the rapid production of ROS and increased expression of IL-2, which exert a feedforward and autocrine effect on the proliferation of T cells [145,146]. The function of selenium is related to the capacity of the selenium to enhance the expression of the alpha (p55) and/or beta (p70/75) subunits of the growth regulatory lymphokine interleukin-2 receptor (IL-2R), thereby promoting their interaction with interleukin-2 and ultimately increasing the rate at which cells proliferate and differentiate into cytotoxic cells [147,148]. Selenium can enhance the stimulation of Ca^2+^ mobilization in T cells and inhibit the ROS-mediated inhibition of T cell activation [149,150]. Selenium may also eliminate age-related defects in lymphocytes from elderly hosts in response to stimulation via proliferation and differentiation into cytotoxic effector cells [151]. A previous study found that a daily dose of 100 μg of yeast selenium to elderly patients in institutions increased their lymphocyte response to pokeweed mitogen [152,153]. In a case-control study of 32 patients with Crohn’s disease and low concentrations (<80 μg/L) of serum selenium, excessive Th1-cell-mediated immune responses in the colon were significantly inhibited when sodium selenite was administered orally at a dose of 360 μg/d for 8–10 weeks [126]. Such findings suggest that a selenium supplementation enhances T cell viability by promoting the differentiation of CD4+ T cells into T-helper-1 (Th1) cells, leading to higher interferon-gamma and CD40 ligand levels [154,155]. Th1 cells drive the type-1 pathway, effectively defending against intracellular pathogens and stimulating delayed-type hypersensitivity (DTH) skin reactions [156,157]. In a 48 week randomised controlled trial of healthy North American men, volunteers who took 300 μg of high-selenium yeast daily increased their blood selenium concentration by 50% and had a normal DTH skin response, while those who took low-selenium yeast exhibited anergy in DTH skin responses [157]. The same results were found when patients with intrinsic asthma were supplemented with selenium [158].

T follicular helper (Tfh) cells are a specialized subset of CD4 + T cells. They play an essential role in the formation of germinal centres (GCs), which are the site where B cells can differentiate into memory B cells and antibody-secreting plasma cells. GPX4 was confirmed to protect Tfh cells from ferroptosis [159], thus enhancing GC reaction. This explains part of the effect of selenium on B cells. Selenoenzymes are also reported to suppress the activity of cellular 5-lipoxygenase in B cells to protect them from oxidative stress [160]. Insufficient or excessive selenium reduces the number of peripheral B cells and B cells in the spleens of mice [161,162].

### 4.3. Brain Function

**Figure 3:**
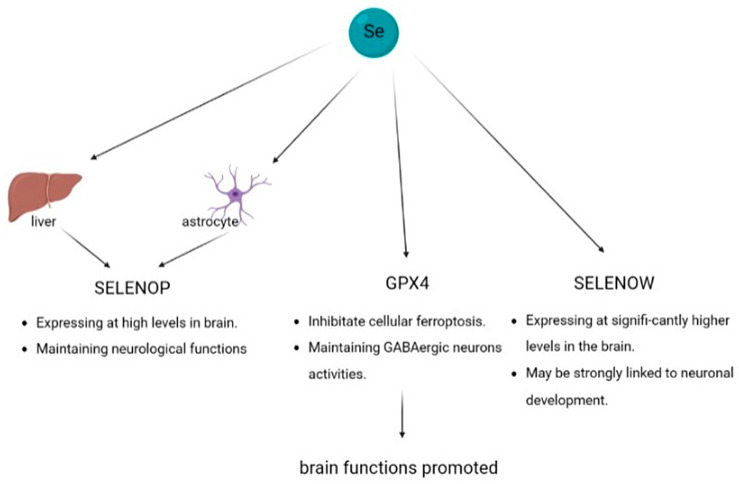
Mechanisms of selenium effects on brain functions. Selenium supplements promote neuronal activities and brain functions by increasing SELENOP, GPX4, and SELENOW levels in brain.

Brain metabolism is highly dependent on selenium levels. The selenium content of the human brain is approximately 90–110 ng/mg wet weight, which is lower than that of the liver. However, when selenium is depleted, brain selenium levels are maintained. These findings demonstrate the significance of selenium in brain function. SELENOP, glutathione peroxidase 4 (GPX4), and SELENOW are the three most highly expressed selenoproteins in the brain, implying that they probably play important roles in brain function (Figure 3). Selenium deficiency can cause irreversible brain injury.

Selenium deficiency can lead to neurological and motor disorders. Plasma selenium levels and erythrocyte GPXs activity are significantly reduced in patients with Alzheimer’s disease (AD) [163,164]. Accordingly, exogenous selenium supplementation has been found to mitigate neurodegeneration and reverse memory deficits in an AD model [165,166]. Parkinson’s disease (PD), a neurodegenerative disease characterised by the dysregulation of motor control, has been found to correlate with selenium levels [167,168]. Selenium reduces bradykinesia in a rat model of PD [169]. Patients with epilepsy usually have lower serum selenium levels than healthy individuals [170,171,172].

#### 4.3.1. Selenoprotein P

The brain can ingest SELENOP from the plasma to acquire selenium. Selenoproteins do not enter the blood–brain barrier and individual cells directly, but are transmembrane via endocytosis of the LRP family receptors, especially LRP8 [173,174]. However, the administration of a selenium-supernutritional diet to Sepp1 knockout mice was found to prevent brain dysfunction, suggesting that plasma SELENOP is not the only route for the brain to obtain selenium, and selenite can be a direct or indirect source of selenium in the brain. Plasma SELENOP levels were almost undetectable in hepatic SELENOP conditional knockout mice, indicating that almost all plasma SELENOP was secreted by the liver. Interestingly, selenium was still incorporated into the brain, and no significant impairment of brain function was observed in SELENOP conditional knockout mice [175]. In contrast, the brain failed to maintain normal function when SELENOP was completely knocked out in mice [176], and the protein levels of two other selenoproteins, GPX4 and SELENOW, were decreased in SELENOP knockout mice. These results confirm that local SELENOP, but not plasma SELENOP, is essential for maintaining brain function, and SELENOP can be stored and recycled in the brain, forming the SELENOP cycle. In addition to being obtained from the blood plasma, astrocytes in the brain may also produce and secrete SELENOP [177].

SELENOP deficiency is associated with neurological deficits and impaired motor functions. It is expressed in over 90% of the brain region at high levels. SELENOP was the first selenoprotein associated with synaptic signaling. Numerous studies have demonstrated that SELENOP promotes neuronal activity and the thalamus, brainstem, and hippocampal neurogenesis are adversely affected when it is under-expressed [17,178,179]. Notably, ataxic manifestations were observed in SELENOP knockout mice [101].

#### 4.3.2. Glutathione Peroxidase 4

GPX4 is a selenoprotein essential for neuronal activity and is found in the nucleus, mitochondria, and cytoplasm. GPX4 is a key molecule in the inhibition of cellular ferroptosis [180,181], and its redox properties contribute to the maintenance of mitochondrial function and inhibition of apoptosis. GPX4 has shown great importance in the brain and in life. Neuronal GPX4-specific knockout was found to be lethal in newborn mice, and was associated with a very high protein abundance during perinatal brain development, which decreased after birth [182]. Neuron-specific inactivation of GPX4 in adult mice leads to massive neurodegeneration, as inhibitory interneurons expressing parvalbumin (PV) are extremely GPX4-sensitive, and PV+ neurons account for 60% of all GABAergic neurons in the somatosensory cortex [183,184]. GPX4 deficiency will make neural activity maintenance difficult and can lead to cerebellar atrophy [185,186]. PD and AD have been found to correlate with GPX4 levels [187,188,189].

#### 4.3.3. Selenoprotein W

Among all selenoproteins, SELENOW was found to be expressed at the highest level in the brain and exhibit representative redox activity. SELENOW is expressed at significantly higher levels in the brain of postnatal mice [190,191], suggesting that it may be strongly linked to neuronal development. Although the exact biological function of SELENOW has not been explored, its high protein levels imply that it may play an important role in the brain.

### 4.4. Cardiovascular System

**Figure 4:**
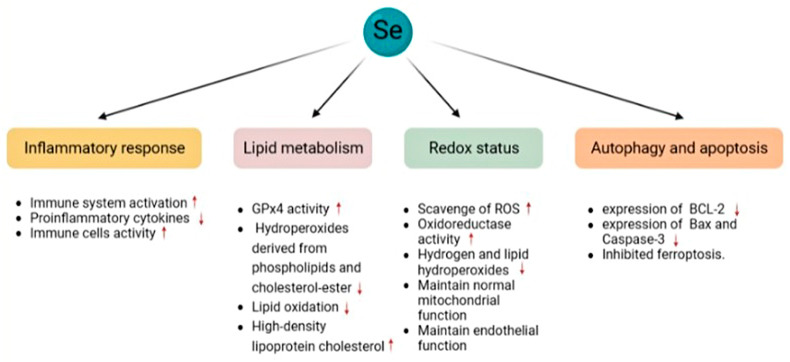
Mechanisms of selenium effects on cardiovascular diseases. Selenium exerts protective effects against CVDs by affecting inflammatory responses, lipid metabolism, oxidative stress, autophagy, and apoptosis in the cardiovascular system.

Selenium is associated with the incidence of CVDs. The earliest evidence of this association is due to Keshan disease, an endemic adolescent disease characterised by cardiomyopathy, which is prevalent in some parts of China with low-selenium soils [192]. Subnormal whole-blood and serum selenium concentrations have been reported in patients with Keshan disease, and selenium supplementation is beneficial for improving this condition [193]. Since then, more cardiovascular effects of selenium have been reported (Figure 4).

In a prior report, plasma selenium concentrations were measured using fluorimetry in 91 hospitalised patients. Based on the results, a significant negative association was found between plasma selenium levels and the severity of coronary atherosclerosis [194]. Animal experiments have also revealed that oral selenium supplementation can reduce the area and degree of atherosclerotic plaques and alleviate vascular inflammation and vascular endothelial dysfunction [195,196,197]; this may be because selenium levels are associated with plasma cholesterol levels. In a double-blind evaluation, selenium supplementation increased serum selenium levels and GPXs activity [198]. GPX4 reduces phospholipids and cholesterol-ester-derived hydroperoxides through GSH, an activator of lipoxygenases and cyclooxygenases, and is necessary for the synthesis of hydroperoxides [199,200]. Therefore, increasing the serum selenium content can reduce lipid oxidation. Similar results were obtained in clinical trials. Plasma lipid peroxidation was reduced by 50% in patients receiving dietary supplementation of 200 g/day selenium compared with the placebo [201]. Increased lipid peroxide concentration due to selenium deficiency may alter prostaglandin synthesis from prostacyclin to thromboxane, resulting in platelet aggregation [202]. Serum selenium levels were found to be positively correlated with the concentration of the high-density lipoprotein cholesterol, which has anti-atherosclerotic effects [198,203].

As a component of selenoproteins, selenium is involved in the regulation of the redox status of cells and participates in the scavenging of ROS and the reduction of hydrogen and lipid hydroperoxides [204]. Therefore, selenium can delay the progression of CVDs and maintain normal cell growth and proliferation, protein folding, and mitochondrial function [181,197]. Selenium can increase the expression and phosphorylation of endothelial nitric oxide synthase to maintain the balance of superoxide anion/nitric oxide and regulate cell adhesion by controlling the expression of cell adhesion molecules, thereby protecting the structural and functional integrity of endothelial cells [205,206]. Selenium can also relieve CVDs by affecting apoptosis and autophagy; increasing the expression of the anti-apoptotic protein BCL-2; reversing the increased expression of the pro-apoptotic proteins Bax and Caspase-3; and regulating the PI3K/AKT/mTOR pathway [207,208,209]. In addition, GPX4 is a key molecule in ferroptosis, and selenium supplementation inhibits ferroptosis [180,210].

In a multinational, prospective, observational cohort study, selenium deficiency was found to be associated with impaired exercise tolerance and a 50% increase in mortality in patients with HF. Researchers have found that selenium is independently associated with impaired mitochondrial function in human cardiomyocytes in vitro [211]. When the case of a patient who died due to cardiomyopathy and ventricular fibrillation was analysed, fatal cardiomyopathy was found to be caused by selenium deficiency. In particular, replacement fibrosis and widespread myocytolysis were observed in the heart [212].

Although few studies found no significant association between selenium and cardiovascular disease [213,214] (for example, an analysis of American physicians found no significant association between plasma levels of the antioxidant selenium and the risk of myocardial infarction [215]), the mainstream notion is that selenium can protect against CVDs and maintain normal cardiovascular function.

### 4.5. Cancer

Based on increasing studies, selenium affects the incidence of cancer. Many cancer cells are selenophilic; however, the selenide, an intermediate product of Sec synthesis, is poisonous. Selenide in cancer cells must be detoxed by selenophosphate synthetase 2 (SEPHS2) [216]. Of note, this process is not required in normal cells. Therefore, when selenium supplementation exceeds a certain dose, selenide accumulates in cancer cells and impairs their growth.

Clinical trials have supported the above conclusion. Early observational studies revealed that individuals with adequate selenium levels in their diet or body tissues have a lower risk of cancer, and plasma selenium levels can decline before some cancers develop [217,218]. However, in a 1973 clinical trial, selenium levels in serum samples collected from 111 patients who developed cancer within the following five years were compared with those from 210 cancer-free individuals matched based on sex, age, and living environment. The findings revealed that the risk of cancer for individuals in the highest quintile of serum selenium was half that of individuals in the lowest quintile [219]. However, some clinical trials have concluded that selenium supplementation does not reduce the overall incidence of cancers, such as lung, bladder, and prostate cancers, with liver cancer as the exception [220,221,222].

We speculate that selenium may confer resistance against cancers when the dosage is appropriate. An eight-year intervention trial was conducted in a general population of 130,471. The incidence of primary liver cancer (PLC) was 35.1% lower in the selenium-supplemented salt group (15 mg sodium selenite per kg) than in the non-supplemented population. After selenium was removed from the treatment group, PLC incidence began to rebound [223]. Selenium protects against breast cancer [224], which has the highest incidence worldwide. A total of 974 men with a history of basal cell carcinoma or squamous cell carcinoma were enrolled in a randomised, double-blind, placebo-controlled trial, receiving 200 μg per day of selenium supplementation or placebo for an average of 4.5 years. At the 6.5 years follow-up, a significant reduction (63%) in the secondary endpoint of prostate cancer incidence was found for men treated with selenium [225]. Similarly, selenium was found to be inversely associated with adenoma and colorectal cancer [226].

The chemical form and bioavailability of selenium, and the stage and type of cancer influence the above results. Most studies on the relationship between selenium and cancer are currently observational studies. As there are still many conflicting conclusions in related studies, further studies are needed to clarify the relevance of selenium in cancer.

### 4.6. Type 2 Diabetes

**Figure 5:**
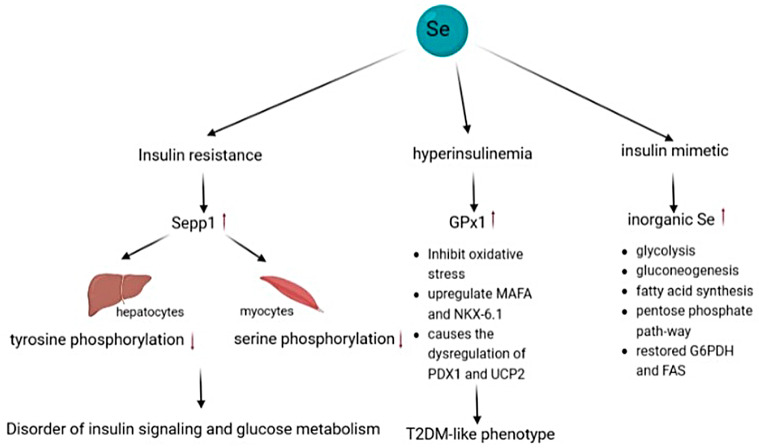
Mechanisms of selenium effects on T2DM. High concentration of Sepp1 induced by high selenium levels impairs insulin signaling and glucose metabolism; and high GPX1 induces T2DM-like phenotype. Inorganic selenium as an insulin analogue aggravates T2DM.

Insulin resistance is a characteristic of type 2 diabetes [227]. The relationship between serum selenium levels and type 2 diabetes mellitus has long been a topic of discussion. Selenium has been shown to affect T2DM through multiple pathways (Figure 5). Most experimental results support a positive correlation between serum selenium levels and T2DM. For example, in a randomised, double-blind, placebo-controlled trial averaging 7.7 years, individuals who took 200 μg/d selenium orally had a higher incidence of T2DM than those who took the placebo [228]. Another dose-response meta-analysis revealed that selenium exposure increased the risk of T2DM as supplementation increased the hepatic production of Sepp1, which is a proven inducer of insulin resistance [229,230,231]. Sepp1 can reduce tyrosine phosphorylation of insulin receptors in hepatocytes, and decrease serine phosphorylation in myocytes, thus impairing their insulin signaling and glucose metabolism [230].

Primary hyperinsulinemia is another pathogenesis of T2DM. Under physiological conditions, the islet β-cells express particularly low amounts of some antioxidant enzymes such as GPXs, catalase and superoxide dismutases; while express moderate to high levels of Sepp1 which exhibits low reactivity with H_2_O_2_; this results in the susceptibility of islet β-cells to ROS. Selenium intake enhances the expression and activity of GPX1. Based on the antioxidant effect of selenium, the upregulation of GPX1 could reduce intracellular H_2_O_2_ production and inhibit islet inflammation and oxidative stress, thereby playing a protective role in islet β-cells [232,233,234]. The formation of insulin is accompanied by the constant formation of disulphide bonds, a process that is susceptible to the redox state. In addition, GPX1 upregulates the transcription factors involved in insulin synthesis, such as MAFA and NKX-6.1. However, these changes are not necessarily beneficial. The overexpression of GPX1 caused by a high selenium status causes the dysregulation of PDX1 and UCP2, and can easily develop into hyperinsulinaemia, decrease insulin sensitivity, and induce the development of a T2DM-like phenotype. Insulin-like effects of high doses of sodium selenomethionine and sodium selenite have been observed in diabetic animals, as in [235,236]. The symptoms of type 2 diabetes were found to be relieved in GPX1 knockout and dietary selenium-deficient mice.

Early studies found that inorganic selenium can act as an insulin mimetic. Selenate was efficient in stimulating glucose ingestion both in vitro and in vivo [237,238] it mimics insulin in glycolysis, gluconeogenesis, fatty acid synthesis, and the pentose phosphate pathway. The expression of glucose-6-phosphate dehydrogenase (G6PDH) and fatty acid synthase (FAS) in rats’ hepatocytes or diabetic animals was restored under the treatment of selenate, suggesting that selenate can stimulate adipogenesis in the liver [239,240]. In 1990, high doses of selenate were found to enhance insulin-stimulated phosphorylation of tyrosine phosphoprotein and insulin receptor kinase activity in rat adipocytes [237].

A meta-analysis of 13,460 individuals revealed that people with relatively lower serum selenium levels (<97.5 μg/L) and relatively higher serum selenium levels (>132.5 μg/L) had a higher prevalence of T2DM. However, the increase in incidence was more obvious in individuals with high selenium levels. In a few other studies, plasma selenium concentration was found to be significantly lower in patients with diabetes than in controls. Such findings suggest that a simple linear relationship does not exist between T2DM and selenium levels, and both high and low selenium levels are potential risk factors for T2DM [241].

### 4.7. Heavy Metal-Based Illness

**Figure 6:**
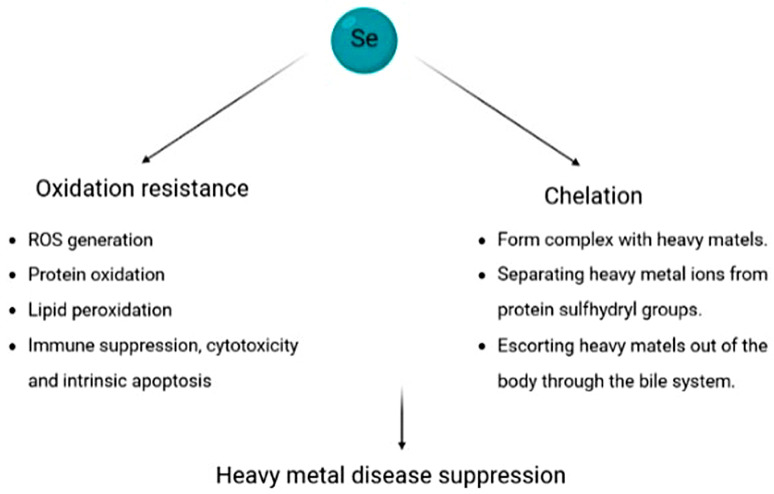
Mechanisms of selenium effects on heavy-metal-based illness. Oxidative stress is the primary toxic mechanism of heavy metals. Selenium detoxifies heavy metal ions by reducing the production of ROS; it can also compete directly with protein sulfhydryl groups for heavy metal ions and excrete them out of the body.

Selenium has been shown to impact oxidation resistance and chelation to inhibit heavy metal toxicity (Figure 6) such as mercury (Hg), cadmium (Cd), arsenic (As), chromium (Cr), thallium (Tl), lead (Pb), and silver (Ag). Metal ions exist in numerous proteins and are required for electron transfer, oxygen transport, catalysis, and other biological processes. However, the accumulation of heavy metals in organisms will induce multiple adverse effects in vivo such as hepatorenal and renal toxicity, neurotoxicity, reproductive toxicity, and immunotoxicity and lead to serious health problems [242,243]. Oxidative stress is the primary toxic mechanism of heavy metals. It is reported that H_2_O_2_ and superoxide anion were dose-dependently increased in mercury-treated erythrocytes [244]. ROS induced by mercury results in both cell necrosis and apoptosis [245]. The liver and kidneys are extremely sensitive to the toxic effects of cadmium. Cadmium is unable to generate ROS by itself; however, it can replace the iron and copper from cytoplasmic and membrane proteins, contributing to the increasing concentration of unbound iron and free copper [246]. They participate in causing oxidative stress via Fenton reactions and impair the mitochondrial electron transport chain and the function of NADPH oxidase [247]. Cadmium atoms can also combine with selenium atoms and lead to a decrease in the synthesis of selenoenzymes. ROS generation and DNA damage induced by arsenic cause a shift in the cell cycle [248]. Chromium causes oxidative damage and a wide range of DNA lesions in the presence of cellular reductants. Mitochondrial dysfunction and cellular deregulation were reported in hippocampal neurons treated with thallium [249]. The results of a meta-analysis manifested that lead treatment causes severe oxidative stress and testicular tissue was more sensitive to lead than other tissues [250]. Silver-mediated dysfunction of the respiratory chain increases the production of ROS [251].

As previously mentioned, selenium functions in the form of selenoproteins, which contribute for antioxidant defense. Both the GPXs and Trx system, which are most important to oxidative defense, are the targets of heavy metal compounds. Selenium supplementation diminished ROS generation, protein oxidation, and lipid peroxidation induced by heavy metals through maintaining the activities of selenoenzymes. Furthermore, it also protects cells from immune suppression, cytotoxicity, and intrinsic apoptosis [150,252,253,254]. The latter probably much relies on its scavenging effect on ROS.

Chelation therapy remains a main treatment for heavy metal poisoning in clinic. Selenium can also interact directly with heavy metals, especially mercury, cadmium, and arsenic, which are usually highly affiliative for sulfhydryl groups and can result in the structural distortion of proteins. However, selenium seems to have higher affinity with heavy metals and can sequester metal ions to reduce their biological availability. It is confirmed that the affinity of mercury for selenium is up to one million times higher than that for sulphur in analogous forms. Selenium was found to form a complex with cadmium or arsenic and escort them out of the body through the bile system [255]. However, cadmium can also undermine the anticarcinogenic effects of selenium (such as liver cancer, renal carcinoma, and prostate cancer) at higher exposures [256,257,258].

## 5. Discussion

The effects of selenium on health are complex. While many novel selenoproteins been identified, their associations with diseases need to be defined. However, to date, no fully unified conclusions have been reached. In this review, we focus on the functions and mechanisms of selenium and selenoproteins as well as their roles in systemic diseases. By summarizing the effects of selenium and selenoproteins on a variety of different diseases, it is not difficult to conclude that selenium supplementation may play a dual role as it exerts anti-inflammatory and antioxidant effects at nutritional doses but reverses these effects at supernutritional doses.

In the future, more specific studies are needed to clarify the mechanisms underlying the effects of selenium on various systemic diseases to determine the appropriate level of supplementation. As the baseline selenium levels of individuals in different populations are not the same, separate studies are required for different populations, in addition to different diseases. The specific molecular mechanisms underlying the effect of selenium supplementation on a particular disease should also be clarified considering the differences between different forms of selenium supplementation. More relevant basic and clinical studies are expected to maximise the benefits and reduce the potential risks of the trace element selenium.

## Figures and Tables

**Table 1:** The functions of 25 selenoproteins and their health effects.

	Function	Health Effects
GPX1	Antioxidant activity; reduce cellular H_2_O_2_.	Cancers [46]; chondrogenic differentiation [47]; T2DM [48]; depression [49]; Keshan disease [50]; cataracts [51]; macular degeneration [52].
GPX2	Antioxidant activity, protect the mucosa of the gastrointestinal tract and various endothelial cells from oxidative damage.	Cancers [53]; intestinal inflammation [54].
GPX3	Reduce lipid hydro peroxides and H_2_O_2_.	Cancers [55]; myocardial fibrosis [56]; ventricular remodeling [57].
GPX4	Antioxidant activity; decrease phosphatidylcholine hydroperoxide; suppress cellular ferroptosis.	Osteoarthritis [58]; cancers [59]; cardiomyopathy [60]; ischemia-reperfusion injury [61]; brain function [62].
GPX6	Not known.	Huntington’s disease [63].
TXNRD1	Antioxidant activity; regenerate thioredoxin; suppress cell ferroptosis.	Idiopathic pulmonary arterial hypertension [64] hepatocellular carcinoma [65]; osteoarthritis [66]; genetic generalized epilepsy [67]; Keshan disease [50].
TXNRD2	Regenerate thioredoxin; regulate cell proliferation and apoptosis.	Primary open-angle glaucoma [68]; CVDs [69,70]; cancers [71]; glaucoma [72].
TXNRD3	Antioxidant activity; suppress pyroptosis.	Male reproduction [73]; colitis and carcinogenesis [74].
DIO1	Activate T3.	Thyroid hormone metabolism [75]; inhibit hepatosteatosis [76]; renal cancer [77].
DIO2	Activate T3.	Osteoarthritis [78]; obesity [79]; mental retardation [80].
DIO3	Inactivate T3.	Osteoarthritis [81]; brain development [82]; sepsis and septic shock [83].
MSRB1	Antioxidant activity; anti-inflammatory effect; regulate immune responses.	Hepatocellular carcinoma [84]; inflammatory response [85].
SEPHS2	Sec synthesis.	Cancers [86].
SELENOF	Immunomodulation; regulate glycogenolysis and lipogenesis; participate in vitamin A metabolism.	Cancers [87]; glucose metabolism disorder [88].
SELENOH	Regulate cell cycle progression and proliferation.	Colorectal cancer [89].
SELENOI	Critical enzyme in the central nervous system; T cell activation; neural development; plasmalogen maintenance.	Hereditary spastic paraplegia 81 [90].
SELENOK	Oxidation resistance; Ca2+ flux regulation; immune regulation; apoptosis regulation; suppress cellular ferroptosis.	AD [91]; cervical cancer [92].
SELENOM	Glucose metabolism; Ca2+ flux regulation; apoptosis regulation.	Glioblastoma [93]; non-alcoholic fatty liver disease [94]; synaptic deficits and cognitive dysfunction [95].
SELENON	Muscle development; calcium haemostasis.	Myopathies [96].
SELENOO	Not known.	Thyroid cancer [97].
SELENOP	Antioxidant activity; maintain neuronal activity; transport selenium to tissues; regulate pancreatic β cell function.	Cancers [98,99,100]; seizures and ataxia [101]; CVDs [102].
SELENOS	Regulate inflammation; induce ER stress apoptosis; immune regulation.	Hashimoto’s thyroiditis [103]; CVDs [104].
SELENOT	Promote nerve regeneration; Ca2+ flux regulation; apoptosis regulation; maintain endoplasmic reticulum homeostasis; regulate glucose and lipid metabolism.	Glioblastoma [105]; AD [106]; CVDs [107].
SELENOV	Regulate glucose and lipid metabolism; prevent endoplasmic reticulum stress and oxidative injury; maintain male reproduction.	Not known.
SELENOW	Oxidation resistance; regulate bone metabolism; support erythroblast development; muscle development.	Osteoporosis [108]; anemia [109].

## Data Availability

Not applicable.
